# Rhizosphere microbiome dataset of Robusta coffee (Coffea canephora L.) grown in the Central Highlands, Vietnam, based on 16S rRNA metagenomics analysis

**DOI:** 10.1016/j.dib.2022.108106

**Published:** 2022-03-28

**Authors:** Dinh Minh Tran

**Affiliations:** Institute of Biotechnology and Environment, Tay Nguyen University, Buon Ma Thuot, Dak Lak 630000, Vietnam

**Keywords:** Rhizosphere microbiome data, Robusta coffee, 16S rRNA metagenomics, The Central Highlands region

## Abstract

Vietnam is the second-largest coffee producer in the world after Brazil. Of the two main coffee production species, namely, Arabica and Robusta, Vietnam is the largest producer of Robusta worldwide [1]. Based on previous reports, the planted coffee area in Vietnam was 695.600 ha and its production was 1.76 million tons in 2020, in which the Central Highlands region accounts for approximately 73% of the planted area and production [2]. Hence, this region is known as the capital of coffee plantations and production in Vietnam. Previous studies have focused on the diversity of rhizospheric bacteria from this plant species cultivated in this region based on cultivation methods [3], [4], [5], [6], [7], [8]. However, no report has been found on the rhizospheric microbial diversity of this important plant in Vietnam. To our knowledge, a dataset of rhizospheric microbial communities of the coffee plant grown in the Central Highlands is still unclear.

This report presents a dataset of the rhizosphere microbiome from a representative sample obtained by mixing five rhizospheric soil samples of Coffea canephora L. cultivated in the Central Highlands region using metagenomic next-generation sequencing. This dataset provides information on the rhizospheric microbial diversity of Robusta coffee, particularly its functionality. Therefore, cultivation techniques for sustainable Robusta coffee production in the region could be developed by applying indigenous rhizospheric microbial resources.

## Specifications Table

**Table:** 

Subject	Microbiology: *Microbiome*
Specific subject area	Metagenomics
Type of data	Figures, Tables, and Fastq files
How the data were acquired	Illumina MiSeq platform
Data format	Raw and Analyzed
Description of data collection	A representative rhizospheric soil sample (500 g) of Coffea canephora L. was mixed from five samples (100 g each) collected from a 6-year-old coffee field in Khanh Xuan Ward, Buon Ma Thuot City, Dak Lak Province. Total microbial genomic DNA was extracted from the sample and the 16S rRNA metagenomic sequencing was performed using the Illumina MiSeq platform (2 × 150-bp paired ends)
Data source location	• Ward/City/Province: Khanh Xuan/Buon Ma Thuot/Dak Lak • Region: The Central Highlands • Country: Vietnam • Latitude and longitude coordinates for collected samples: 12°38′55.20′′N,107°59′22.06′′E
Data accessibility	Data are available at the NCBI with Bioproject PRJNA797920 (https://www.ncbi.nlm.nih.gov/Traces/study/?acc=PRJNA797920)

## Value of the Data

• Data provide information on the rhizospheric microbial diversity of Coffea canephora L. and its functionality in the Central Highlands and other regions in Vietnam.
• Data could be used for the comparative analysis of the rhizospheric and endophytic microbiome profiles of Coffea canephora L. cultivated in the Central Highlands and other regions in Vietnam.
• Data will be useful for subsequent studies on the conservation of rhizospheric microbial genetic resources and the development of cultivation techniques for applying them for sustainable Robusta coffee production in the Central Highlands to achieve the nutrients required for various stages of development and growth.

## 1 Data Description

**Fig. 1:**
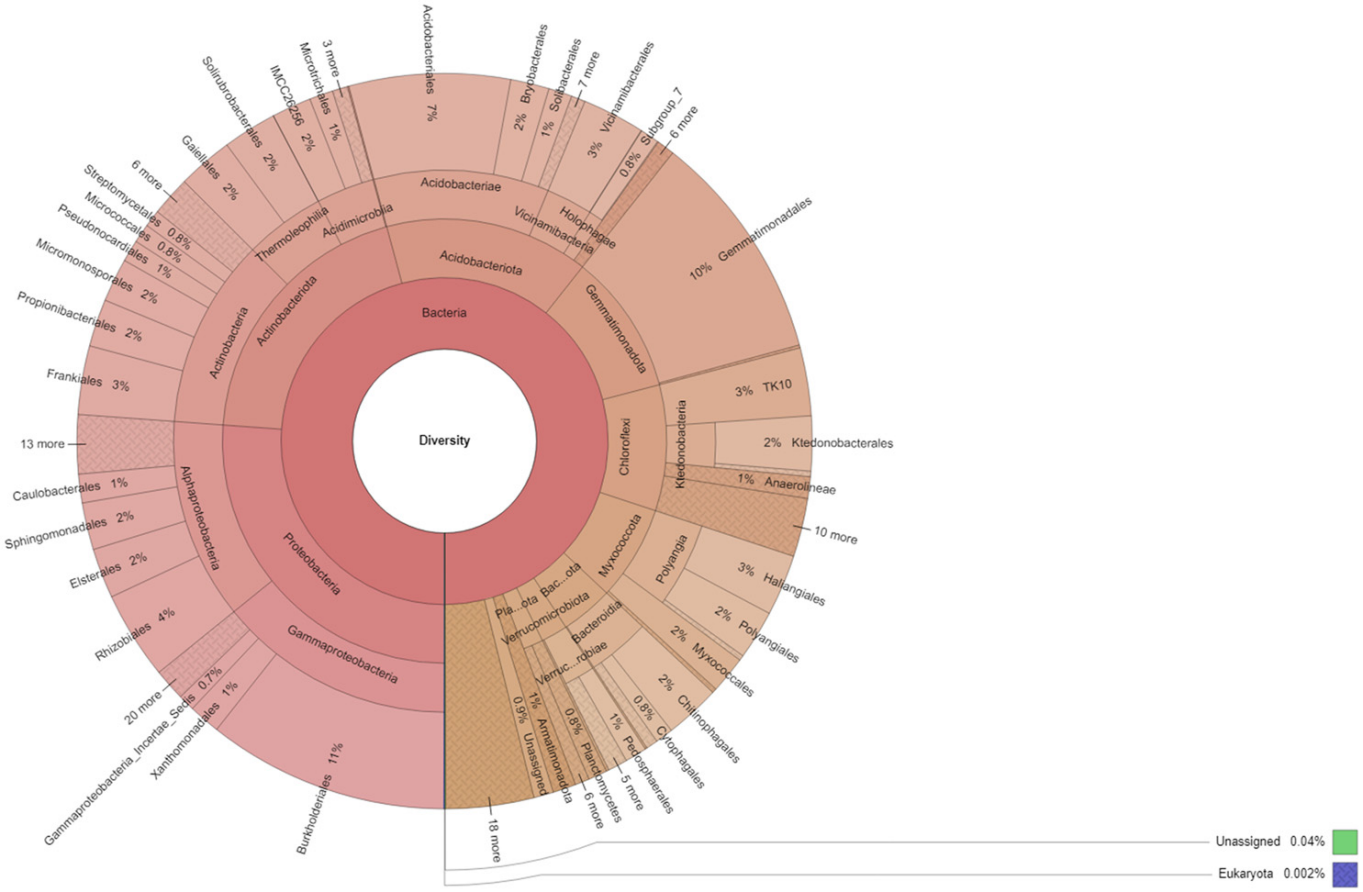
Taxonomic profiles of rhizosphere microbiome of Coffea canephora L. in the Central Highlands region, Vietnam.

This dataset presents the taxonomic and functional profiles of the rhizosphere microbiome of Coffea canephora L. cultivated in the Dak Lak Province of the Central Highlands, Vietnam. The results showed that 256,357 reads were identified from 256,462 analyzed reads (Table 1). Taxonomic analysis (Fig. 1) showed that 28 phyla were identified from the sample. Among these phyla, Proteobacteria (26.4%) were the most abundant, followed by Actinobacteriota (19.83%), Acidobacteriota (15%), Gemmatimonadota (10.35%), Chloroflexi (9.24%), and Myxococcota (6.97%). Of the 119 bacterial orders detected, Burkholderiales (10.74%) were shown to be the most dominant, followed by Gemmatimonadales (10.22%), Acidobacteriales (7.15%), Rhizobiales (3.91%), and Frankiales (3.04%). Moreover, 156 families were detected. Among these families, Gemmatimonadaceae (10.22%) were found to be the most abundant, followed by Xanthobacteraceae (2.96%), Haliangiaceae (2.71%), Nitrosomonadaceae (2.56%), and Chitinophagaceae (2.44%). Finally, 242 genera were identified from the rhizosphere of Robusta coffee.

**Table 1:** Summary of analyzed, classified, and unclassified reads in this study.

Reads	Count
Total analyzed reads	256,462
Classified reads	256,357
Unclassified reads	105

**Fig. 2:**
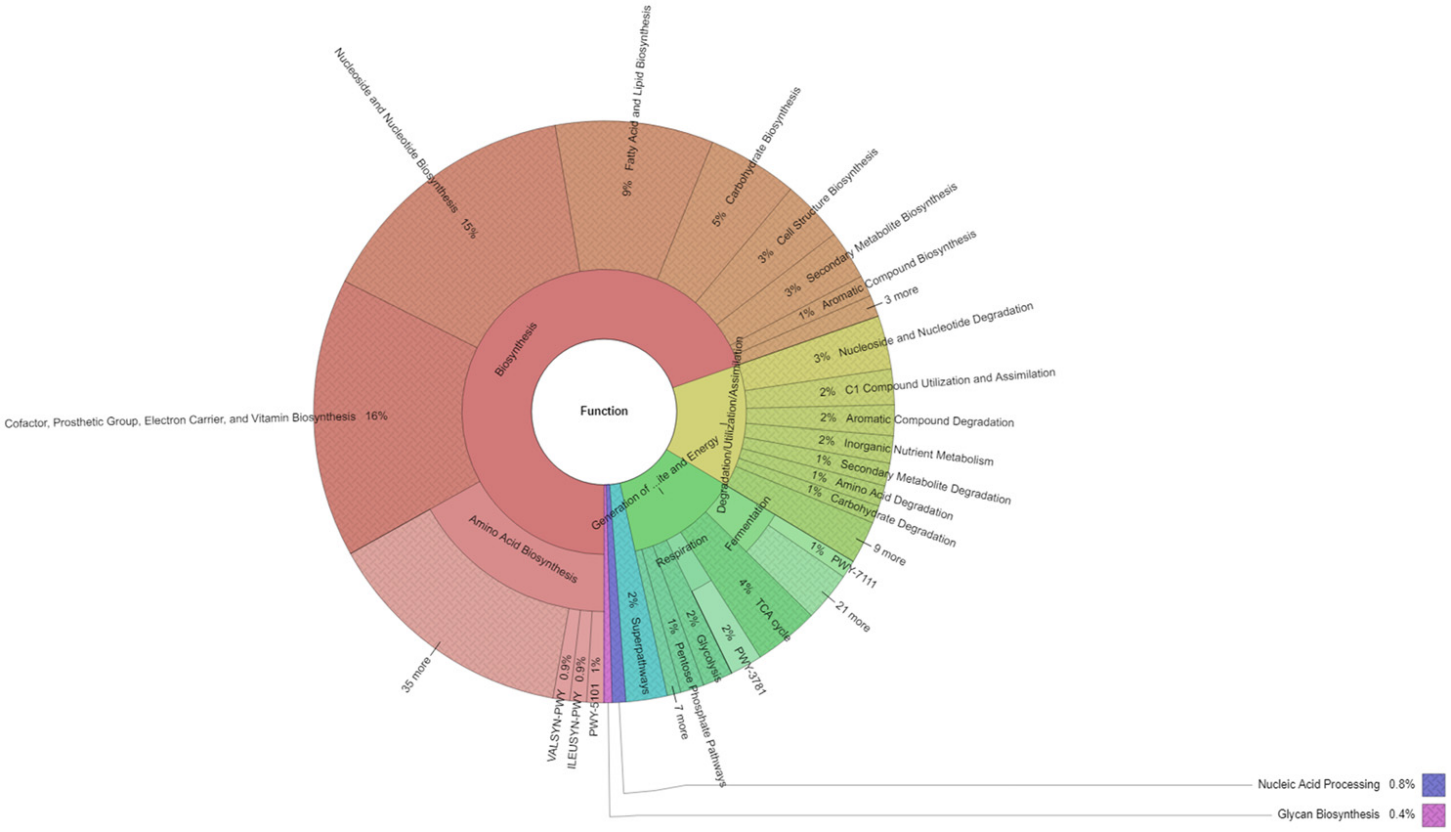
Functional profiles of rhizosphere microbiome of Coffea canephora L. in the Central Highlands region, Vietnam.

Functional analysis (Fig. 2) showed that the primary function of the microbiome of Robusta coffee was biosynthesis (69.67%), followed by degradation/utilization/assimilation (13.92%) and the generation of precursor metabolites and energy (12.92%). Among the functions involved in biosynthesis, amino acid biosynthesis (16.91%) was the most abundant, followed by cofactor, prosthetic group, electron carrier, and vitamin biosynthesis (15.51%); nucleoside and nucleotide biosynthesis (14.88%); fatty acid and lipid biosynthesis (8.8%); carbohydrate biosynthesis (5.02%); cell structure biosynthesis (3.43%); and secondary metabolite biosynthesis (2.75%).

## 2 Experimental Design, Materials and Methods

### 2.1 Rhizospheric soil sampling

Five rhizospheric soil samples (approximately 100 g each, 5–30 cm in depth) of Coffea canephora L. were collected from five different sites of a 6-year-old coffee field in Khanh Xuan Ward, Buon Ma Thuot City, Dak Lak Province, on October 30, 2021. The samples were mixed well, combined into one representative sample, kept at 4°C in an ice box, and brought to the laboratory within 1 h after sampling. The sample was stored at −80°C until analysis.

### 2.2 Isolation of microbial genomic DNA

Microbial genomic DNA was extracted from 300 mg of soil sample using the DNeasy PowerSoil kit (Qiagen, Germany) in accordance with the supplier's instructions.

### 2.3 Library preparation and 16S rRNA metagenomic sequencing

The 16S rRNA gene (regions V1–V9) was amplified using primers [9], and then libraries of 16S rRNA gene amplicons were prepared using the Swift amplicon 16S plus ITS (internal transcribed spacer) panel kit (Swift Biosciences, USA) in accordance with the supplier's instructions. Finally, the Illumina MiSeq platform (2 × 150 bp paired ends) was used to perform the library 16S rRNA gene amplicon sequencing.

### 2.4 Taxonomic and functional analyses

Taxonomic and functional profiles of rhizospheric microbes were analyzed in accordance with the method of Tran et al. [9]. In brief, bcl2fastq was used to demultiplex raw basecall files. Adapters, primers, and low-quality sequences (average score of <20 and read length of <100 bp) were removed using Trimmomatic (version 0.39) [10] and Cutadapt (version 2.10) [11]. The q2-dada2 plugin and denoise-single method within the QIIME2 pipeline (version 2020.8) [12] were used to cluster and dereplicate the reads into amplicon sequence variants. QIIME2 aligned with the SILVA SSURef reference database (version 138) [13] was used for taxonomic analysis of amplicon sequence variants in accordance with the classify-consensus-blast method [14]. Finally, PICRUSt2 (version 2.3.0-b) [15] and MetaCyc databases [16] were used to deduce the functional profiles of rhizospheric microorganisms from the sample based on the results of 16S rRNA gene amplicon sequencing.

## Ethics Statements

None

## CRediT Author Statement

**Dinh Minh Tran:** Conceptualization, Methodology, Investigation, Formal analysis, Software, Data curation, Validation, Visualization, Writing – original draft, Writing – review & editing.

## Declaration of Competing Interest

The author declares that it has no known competing financial interests or personal relationships that could have appeared to influence the work reported in this paper.

## Data Availability

Rhizosphere microbiome dataset of the Robusta coffee (Coffea canephora L.) grown in the Central Highlands, Vietnam, based on analysis using 16S rRNA metagenomics (Original data).
